# Evaluating the feasibility and acceptability of an online research capacity-building education programme for the health and care workforce: a mixed-methods pilot study

**DOI:** 10.1136/bmjopen-2026-118946

**Published:** 2026-08-11

**Authors:** Scott Lamont, Kathryn Berzins, Georgia Aspinall, Hayley J Lowther-Payne, Paul Boland, Catherine Harris, Joanna Harrison, Yasemin Hirst, Joseph Spencer, Jo Catherine Weldon, Caroline Leigh Watkins

**Affiliations:** 1 University of Lancashire, Preston, Lancashire, UK; 2 University of Technology Sydney Faculty of Health, Ultimo, New South Wales, Australia

**Keywords:** Health Workforce, Research Design, EDUCATION & TRAINING (see Medical Education & Training), QUALITATIVE RESEARCH, Capacity Building, Evidence-Based Practice

## Abstract

**Abstract:**

**Objectives:**

To evaluate the feasibility and acceptability of an online research capacity-building (RCB) education programme for the health and care workforce and explore its preliminary outcomes.

**Design:**

Mixed-methods pilot study incorporating quantitative survey data and a qualitative group interview.

**Setting:**

Single Health Innovation organisation in England’s North West region.

**Participants:**

Staff working in research, evaluation and innovation-related roles.

**Interventions:**

16 one-hour online RCB education sessions, informed by the Research Capacity and Culture Tool (individual domain), were delivered weekly via Microsoft Teams between February 2024 and October 2024.

**Primary and secondary outcome measures:**

The primary outcomes were feasibility and acceptability of the programme, assessed through attendance and a participant group interview. The secondary outcome was change in behaviour intention measured pre–post education sessions using the Continuing Professional Development Reaction (CPD-R) questionnaire.

**Results:**

Attendance per RCB education session ranged from 2 to 11 participants. Post-session CPD-R completion was low, limiting matched pre–post analysis, but median scores were generally higher post-session across most constructs. Four themes and eight sub-themes were identified from qualitative data. The four core themes were: establishing a common ground for research capacity building; creating pathways for meaningful involvement; translating knowledge within organisational contexts; and bridging individual development and collective momentum. The online format was received well for its accessibility and convenience, fitting within busy working schedules. Participants also reported increased confidence and perceived skill development.

**Conclusions:**

This pilot study demonstrates initial feasibility and acceptability of an online format for RCB education within the health and care workforce. The format has potential for delivery at scale, where time and service pressures limit access to face-to-face programmes. Session length and content support require refinement in future iterations. Further research should examine implementation across diverse settings with larger samples.

**STRENGTHS AND LIMITATIONS OF THIS STUDY**

- The theory-informed research capacity-building (RCB) education programme was evaluated using a mixed-methods design incorporating pre–post quantitative surveys and a qualitative group interview.
- The online format is a novel approach to RCB education, designed for flexible delivery at scale across diverse workforce settings.
- Qualitative analysis involved independent coding by two researchers and member checking with three participants.
- The study was conducted within a single health innovation organisation in the North West of England, limiting transferability to other workforce settings or regions.
- Quantitative findings should be interpreted as indicative only, due to small sample size and low post-session questionnaire completion.

## Introduction

 Research capacity building (RCB) is a key priority for healthcare organisations within a context of emerging evidence and the need for innovation.^1^ However, the health workforce may lack specific knowledge, skills and support to engage in research activities.^2^ Structured RCB initiatives can help address this gap, strengthening organisations’ ability to respond to changing needs while supporting individuals to use evidence in practice and improve health outcomes. The WHO defines RCB, variously referred to as research capacity development or research capacity strengthening, as the capacity to ‘do, manage, share, and apply research.^3^’ The health and social care sectors have seen staff RCB initiatives as a way to enhance their research capabilities and to support organisational planning and delivery.^4^

Two key foundational frameworks have emerged over the last 20 years to support RCB within healthcare contexts. Cooke’s^5^ model identified six principles for effective RCB: skill development, fostering partnerships, aligning research with practice, effective dissemination, infrastructure investment and sustainability. Subsequently, the Research Capacity Development for Impact framework emphasised multi-level interventions that include experiential learning, collaboration and co-production.^6^ The importance of considering RCB at the individual, team and organisational contexts was highlighted in these frameworks.

Research capacity building, while shaped by individual knowledge, skills and motivation, must also be considered in the context of broader organisational issues. For example, leadership ‘buy-in,’ access to research networks and expertise, and internal systems which support research endeavours are all linked to organisational readiness and capacity across professions. However, studies exploring research capacity and culture suggest that organisational backing is often only moderate, while competing workload priorities and limited research skills continue to affect individuals’ ability to participate meaningfully in research activities.^7 8^ Without integrating RCB into everyday organisational structures and policy, progress towards sustainable research cultures is challenging.^9^

Various approaches to RCB exist, primarily involving face-to-face training. These can be seen in internship^10 11^ and fellowship^12 13^ programmes, often requiring staff release or dedicated work–study time which is not always feasible across services. Aimed at developing individual capabilities and organisational capacity for research endeavours,^9^ face-to-face RCB programmes incorporating mentorship and practical training have demonstrated success in building research capabilities in individuals.^14 15^

In the UK, recent strategic guidance within healthcare supports a shift to having a research-ready clinical workforce. This is articulated in the National Health Service (NHS) England Multi-professional Practice-based Research Capabilities Framework, which aims to transform health and care delivery, enhance individual and community health outcomes and experiences, while supporting all health and care professionals to engage in and with research, irrespective of care setting.^16^

Within the current policy landscape, challenges remain in delivering RCB activities to diverse, time-constrained workforces. Online RCB education offers a flexible, scalable alternative to traditional face-to-face RCB programmes. Understanding the feasibility and acceptability of online programmes can inform their future implementation and potential for wider delivery across healthcare settings.

### Aim

To evaluate the feasibility and acceptability of an online research capacity-building education programme and explore preliminary outcomes to inform future scale delivery.

## Methods

### Design

Mixed-methods pilot study incorporating quantitative survey data and a qualitative group interview.

### Context and setting

This study was conducted in collaboration with a Health Innovation organisation in England’s North West region. The organisation is one of 15 regional Health Innovation Networks established by NHS England to connect the NHS, academia, industry, local authorities and the third sector, supporting the adoption and spread of innovation across health and social care. The organisation includes staff from diverse professional backgrounds, including health, science and Information Technology (IT). An online RCB education programme was designed and delivered to staff to develop foundational research skills as part of organisational efforts to strengthen research capacity. Due to service pressures and limited staff capacity to participate in more intensive face-to-face training, the online modular format was developed to provide flexible access. Sessions were designed and facilitated by study authors selected for relevant methodological expertise from within the Applied Research Collaboration North West Coast.

### Theoretical framework

This study was underpinned by two complementary theory-informed frameworks. The Continuing Professional Development Reaction (CPD-R) questionnaire^17^ was used to examine behaviour intention pre-post intervention. Subsequently, Normalisation Process Theory (NPT)^18^ helped explore the conditions influencing implementation within routine work.

### Intervention

16 RCB education sessions were developed (online supplemental file 1). These hour-long sessions focused on 13 of 14 Research Capacity and Culture tool ‘Individual’ items (‘Securing Research Funding’ was excluded following organisational consultation).^7^ An additional two items were added by the research team (Implementation Science and Health Inequalities), as was an Introductory session. The format was developed with the partner organisation around protected weekly CPD time and constraints on releasing staff for face-to-face training. While the RCC tool has previously been used to assess research capacity and identify training needs, its use to structure the content of a delivered education programme is, to our knowledge, novel. The sessions were delivered weekly via Microsoft Teams, with time-limited access to recordings available to those not in attendance. Session slides and follow-up opportunities with facilitators were also available following each session. Sessions were delivered between February 2024 and October 2024 (extended due to cancelled sessions and holiday periods).

### Participant recruitment

Participants were recruited via internal staff email, circulated by a member of the organisation’s evaluation team. Participants were invited to online RCB education sessions, available to staff in roles relevant to research, evaluation and innovation activity. Sessions were available to all eligible staff, and individual attendance across sessions was not tracked, as participation in one session was not a prerequisite for another. Education session attendees were then invited to take part in a group interview exploring perceptions of the programme’s feasibility, acceptability and potential to influence practice.

### Data collection

Quantitative data were collected using pre-post session surveys for each education session. Surveys used the CPD-R questionnaire to examine participants’ behaviour intentions relating to respective sessions. Post-session surveys remained open for 4 weeks, with participants asked to complete them 2–4 weeks after each session to allow time for reflection while retaining session proximity. The CPD-R comprises 12 items across five constructs: Intention, Social Influence, Beliefs about Capabilities, Moral Norm and Beliefs about Consequences (online supplemental file 2). Surveys were completed online via the Qualtrics platform, and consent was assumed by completion and return. Qualitative data were collected through a group interview following programme delivery. Guided by NPT, the interview explored participants’ perceptions of the programme and the conditions influencing its use in practice (table 1). The group interview was conducted online via Microsoft Teams by two researchers from an applied health research team (GA, HJL-P) who were undertaking postgraduate research degrees, had experience in qualitative methods and were independent of the education delivery. The interview was recorded and transcribed for analysis.

**Table 1:** Group interview guide mapped to NPT domains

NPT domain	Questions
Coherence (making sense of the intervention)	What did you initially understand about the purpose and goals of the sessions? How did you understand the purpose of the sessions in relation to your current role? How did the content and structure of the sessions align with your understanding of research capacity building?
Cognitive participation (engagement with the intervention)	How engaged did you feel during the sessions? Were there any moments you felt particularly motivated or disengaged? How did interactions with other participants or facilitators influence your learning? Which specific topics or sessions facilitated the practical application of new knowledge, and how?
Collective action (enacting the intervention)	How have you integrated the research skills or attributes into your daily work practices? What factors have enabled or hindered your ability to apply these research skills? Were there any aspects of the sessions that you found challenging or difficult to understand?
Reflexive monitoring (appraising the intervention):	How have the sessions changed your approach to research? and how would you evaluate their effectiveness in enhancing research capabilities of yourself and colleagues? Do you feel that the sessions met your expectations? Why or why not?

NPT, Normalisation Process Theory.

### Data analysis

Quantitative data from the pre-post education session surveys were analysed by the first author (SL—mixed-methods researcher) using descriptive statistics, with median scores calculated to assess behaviour intention. Qualitative data from the group interview followed six phases of thematic analysis described by Braun and Clarke.^19^ Two researchers (GA, HJL-P) independently coded data and generated initial themes, with a third researcher (SL) assisting in refining and finalising themes. Analysis was guided by and mapped to NPT constructs (Coherence, Cognitive participation, Collective action and Reflexive monitoring) to identify barriers and enablers to applying research skills in practice. To enhance trustworthiness, member-checking was conducted, where three group interview participants responded to a request and confirmed the accuracy and credibility of the themes identified. CPD-R data offered preliminary, exploratory insight into behavioural intention. Qualitative findings addressed both feasibility and acceptability, including practical and logistical factors relevant to delivery. These provided complementary rather than convergent evidence across the study.

### Patient and public involvement

A public advisor working within the Applied Research Collaboration North West Coast contributed to the co-design of the health inequalities education session within the programme. No patients or members of the public were involved in the study design, data collection or analysis. Public involvement is planned as part of knowledge mobilisation activities.

### Ethical approval

The study received ethical approval from the University of Lancashire Ethics Review Panel (HEALTH 01076 FR). All data were securely stored on a university-hosted secure server in accordance with General Data Protection Regulation (GDPR) requirements and the Data Protection Act 2018. Survey participants were informed via a participant information sheet that consent was implied by completion and submission of the survey. Group interview participants submitted written consent forms to the lead author before the interview, and consent was verbally reaffirmed by the facilitators at the start of the group interview.

## Results

Participation in the education sessions varied, with attendance ranging from 2 to 11 participants per session. Completion rates for the pre-session CPD-R questionnaires were generally commensurate with attendance, but the post-session questionnaire completion was consistently lower across all sessions, ranging from 1 to 6. Thus, the number of usable matched surveys pre-post session was low. Due to the small number of participants and the low response rates for matched pre-post-session questionnaires, we were unable to perform inferential statistical analysis to assess the significance of observed changes. Accordingly, the CPD-R findings are presented descriptively and should be interpreted as exploratory. Median scores were generally higher post-session across most constructs, with directional increases in participants’ beliefs about capabilities, moral norm and beliefs about consequences. Although shifts in social influence were generally more modest, some sessions showed increases in median social influence scores.

Larger increases in median scores were observed in beliefs about capabilities for most sessions, notably in sessions such as using a computer referencing system, writing a research protocol and submitting an ethics application. Similarly, moral norm and beliefs about consequences most often showed increases in median scores or remained high across sessions. Sessions such as finding relevant literature and analysing quantitative research data showed larger increases in median scores across intention, beliefs about capabilities and beliefs about consequences post-session. Completing a health inequalities assessment tool and understanding implementation science also showed increases in median scores across all five constructs, although these were based on very small numbers of matched responses (1–2 observations). In contrast, changes in social influence were generally more modest across the sessions, reflecting less pronounced shifts in participants’ perceptions of peer behaviour or external expectations (see table 2 for CPD-R pre-post-test medians).

**Table 2:** CPD-R questionnaire results (matched pre-post responses only)

	Pre-test median	Post-test median		Pre-test median	Post-test median
**Finding relevant literature (n=5**)			**Analysing qualitative research data (n=5**)		
Intention	5.5	6.0	Intention	6.3	6.8
Social influence	2.7	4.0	Social influence	3.4	4.0
Beliefs about capabilities	3.7	6.3	Beliefs about capabilities	4.9	6.0
Moral norm	5.5	7.0	Moral norm	7.0	7.0
Beliefs about consequences	6.0	7.0	Beliefs about consequences	7.0	7.0
**Critically reviewing the literature (n=4**)			**Analysing quantitative research data (n=3**)		
Intention	5.5	6.0	Intention	5.0	7.0
Social influence	3.2	3.0	Social influence	3.3	4.0
Beliefs about capabilities	4.9	5.9	Beliefs about capabilities	4.7	6.0
Moral norm	6.5	6.8	Moral norm	7.0	7.0
Beliefs about consequences	6.5	6.0	Beliefs about consequences	6.0	7.0
**Using a computer referencing system (n=6**)			**Writing a research report (n=2**)		
Intention	4.0	6.0	Intention	6.5	6.8
Social influence	1.7	2.3	Social influence	3.7	2.2
Beliefs about capabilities	2.3	6.0	Beliefs about capabilities	4.7	6.0
Moral norm	3.0	6.0	Moral norm	6.5	7.0
Beliefs about consequences	3.5	5.0	Beliefs about consequences	6.5	6.5
**Writing a research protocol (n=6**)			**Writing for publication in peer-reviewed journals (n=1**)		
Intention	5.5	6.5	Intention	5.5	6.0
Social influence	2.3	4.7	Social influence	4.0	5.0
Beliefs about capabilities	4.3	6.0	Beliefs about capabilities	4.0	5.3
Moral norm	6.0	7.0	Moral norm	7.0	7.0
Beliefs about consequences	6.0	7.0	Beliefs about consequences	7.0	7.0
**Submitting an ethics application (n=5**)			**Supporting less experienced researchers (n=2**)		
Intention	6.0	7.0	Intention	5.0	6.5
Social influence	2.0	3.7	Social influence	3.7	5.7
Beliefs about capabilities	4.7	6.3	Beliefs about capabilities	4.7	5.7
Moral norm	5.5	7.0	Moral norm	6.0	7.0
Beliefs about consequences	6.0	7.0	Beliefs about consequences	5.0	7.0
**Designing questionnaires (n=4**)			**Understanding implementation science (n=2**)		
Intention	7.0	7.0	Intention	5.5	6.5
Social influence	3.7	4.5	Social influence	2.7	3.7
Beliefs about capabilities	6.3	6.7	Beliefs about capabilities	4.2	6.0
Moral norm	6.8	7.0	Moral norm	6.0	7.0
Beliefs about consequences	7.0	7.0	Beliefs about consequences	5.3	6.5
**Collecting data (n=4**)			**Completing a health inequalities assessment tool (n=2**)		
Intention	7.0	6.8	Intention	5.8	6.8
Social influence	4.0	3.8	Social influence	4.7	5.2
Beliefs about capabilities	5.7	6.7	Beliefs about capabilities	5.0	6.2
Moral norm	6.5	7.0	Moral norm	6.3	7.0
Beliefs about consequences	7.0	7.0	Beliefs about consequences	6.3	7.0
**Using computer data management systems (n=3**)					
Intention	4.0	4.5			
Social influence	3.0	3.3			
Beliefs about capabilities	4.0	5.0			
Moral norm	7.0	7.0			
Beliefs about consequences	4.0	5.0			

CPD-R, Continuing Professional Development Reaction.

### Group interview

Seven participants joined the group interview. Among them were four project/programme managers, along with a project support officer, a business intelligence officer and a biomedical scientist. Their backgrounds in research ranged from formal training to more hands-on, practical expertise. Four main themes emerged from the analysis; each connected to a construct of the NPT framework. These themes are broken down into sub-themes to provide nuance. The findings reflect factors that supported or hindered how participants engaged with the RCB education and used subsequent learnings in their work.

## Coherence

### Theme 1: establishing a common ground for research capacity building

Strengthening existing knowledge, developing new research skills and addressing practice challenges reflected how participants understood and made sense of the RCB education sessions. A shared sense of purpose appeared to support alignment around the programme’s aims, important for fostering collaboration. Challenges were noted in adapting and translating some content into day-to-day activities, highlighting the need to understand who the intervention is for and what relevant opportunities exist to apply learning.

#### Sub-theme 1.1: unified vision across roles

Participants considered the sessions as opportunities to build research skills among the team to align with organisational goals. A shared aim helped build a sense of team cohesion in preparation for collaborative working:

I thought the purpose of it was partly about upskilling the team in general and to make sure we were all on the same page (P5).

while another noted:

We’ve realised that we’ve got loads of skills across the organisation, but maybe people have got some gaps in their knowledge (P3).

#### Sub-theme 1.2: personal meaning and professional fit

An appreciation of best practices in research application and how this aligns with role responsibilities at the individual level was discussed. One notable example was provided by a participant involved in evaluation work:

So it’s been nice to know what to do, what to avoid and what could make the evaluation better (P6).

This was not the case for all participants, highlighting that diversity among the group in relation to job specifications challenged the uniform tailoring of the sessions:

Some of the things were useful for my job, but others were more for traditional research roles and harder to apply (P4).

## Cognitive participation

### Theme 2: creating pathways for meaningful involvement

When participants perceived the content of RCB sessions as relevant and applicable to daily activities, how they engaged with sessions was enhanced. They appreciated the interactive elements of the sessions, as well as the accessibility of session resources such as slides, recordings and follow-up opportunities with facilitators. However, limited applicability of certain topics, an absence of practical examples in some sessions, and limited time for discussion at the end of some sessions were highlighted.

#### Sub-theme 2.1: resonance through practical relevance

Participants were more engaged when content connected clearly to their everyday responsibilities. One described this simply:

So the things that were more useful for me were things that were relevant to my job (P4).

Although acknowledged as potentially beneficial, less immediately applicable learning had a weaker impact on engagement:

But there were some of the modules that we’re just not using day-to-day, but that’s not saying in the future they won’t be useful and actually knowing what you don’t know is really helpful as well (P3).

#### Sub-theme 2.2: facilitating learning through interaction and support

Group discussion, access to slides and recordings post-session, and the option to follow-up with facilitators contributed to a greater sense of interaction and engagement with the sessions:

I think the discussion bit at the end is probably the most useful (…) those sessions that went over would probably either need more time or, you know, be shortened (P5).

while another noted:

It was helpful with the slide sets though; I think pretty much everyone apart from one left contact emails, so at least we could go back to them and ask for further information (P4).

Building on this, one participant also emphasised the importance of interactive tools to maintain focus and engagement during sessions, which they had experienced in a different training programme:

My attention span’s horrific these days… what they did was they had quite regular Slido questions just to check that you were understanding and on a smaller subject, it’s probably doable just to take a minute here or there just to make sure that people are… actually taking it in. (P2).

Participants appreciated having time to engage with session content, especially when it was dense or complex. But that wasn’t always possible:

Some sessions ran over, so we didn’t always get to discuss things properly (P5).

## Collective action

### Theme 3: translating knowledge within organisational contexts

How the sessions’ content was enacted and applied within daily routines was enhanced when actionable opportunities existed for participants, but barriers were present with some content that participants found complex or less aligned with specific professional roles.

#### Sub-theme 3.1: applying insights in practice

Participants reported applying learning from sessions in several ways, including work on questionnaire design, service evaluations and navigating ethics approvals. One participant gave an example of applying these skills in a recent reporting project:

We’ve just published or are just publishing a report on (…) and it’s really clear from both the way that we did the work and the write-up (…) that we have applied what we’ve been learning over the last year into that (P5).

One participant described how both the sessions and the researchers supported the success of a recent project:

Everyone has been commenting on how fantastic the project was (…) the session and the researchers were instrumental in making sure that the project was a success (P6).

These accounts suggest participants perceived the sessions as having practical relevance to their work, supporting the acceptability of the programme.

#### Sub-theme 3.2: aligning learning with contextual realities

Although many participants were able to apply their learning, some pointed to practical limitations. For a few, the chance to use new skills hadn’t yet come up, which made it harder to embed the learning in day-to-day work.

I’ve not had as much opportunity to apply what I’ve learned from the sessions I attended (P1).

In addition, content considered as complex may require deeper exploration to fully grasp and apply:

The quantitative stuff… I think that one needed a bit more space to allow it to absorb and go through it slowly (P7).

## Reflexive monitoring

### Theme 4: bridging individual development and collective momentum

Changes in how participants approached research following the RCB sessions reflected an increase in research-related confidence and ability to engage with academic partners. A need for structured processes which supported evaluation and sustainment of learning and new skills was also highlighted, particularly across teams with varied responsibilities.

#### Sub-theme 4.1: confidence as a foundation for action

Feeling more able to work with academic ideas and assess their own practice came through in several participants’ accounts:

I think it’s given me confidence to have the conversations with people who are proper academics, for want of a better phrase (P5),

and:

Confidence again, really just making sure that we feel confident that we’re doing the right things and being able to push back at the right times (P3).

#### Sub-theme 4.2: formal mechanisms for sustainable growth

There was uncertainty around how change could be assessed over time. Some pointed to the need for clearer mechanisms to review progress and understand the outcomes of the sessions:

It would make sense wouldn’t it, to see, to track that progress, because we’ve come a long way (P3).

We haven’t planned next steps as a team, which would really help to see how this is affecting us (P1).

I think a proxy measure will be the number of projects we take on, how busy we’ve become, whether we get anything published (P7)*.*

## Discussion

This study evaluated the feasibility and acceptability of an online RCB education programme with preliminary outcomes. The online modular format was well-received for its flexibility and accessibility. Online delivery proved feasible, and short sessions minimised disruption to workloads. Post-session median behaviour intention scores were generally higher across most CPD-R constructs. These scores indicate participant readiness to apply learning in practice; however, low matched responses limit the strength of this interpretation and should be considered alongside the qualitative findings. Qualitative feedback indicated perceived increases in confidence and the application of skills among participants. The online format for RCB education shows potential for wider delivery, as part of broader RCB initiatives and programmes. This may be particularly relevant where workforce capacity limits participation in more resource-intensive RCB initiatives.

Brief, online RCB education may offer a flexible and potentially low-cost approach to delivering learning, particularly where time-pressured constraints exist.^20 21^ Online delivery of research methods education can support research skill development and may improve accessibility for learners unable to attend in person.^22^ More broadly, online learning for healthcare professionals may achieve learning outcomes comparable to face-to-face approaches, while offering flexibility around professional commitments.^23 24^ Participants in this study indicated that for training to be effective, it needs to reflect what participants actually do in their roles. This idea is echoed in the work of Cordrey *et al*^25^ and Brandenburg *et al*,^26^ who found that content is more effective when tailored to the distinct needs of specialist teams. At a broader level, organisational alignment has also been linked to more sustainable research capacity. The ongoing tension between individual and organisational aims found here reflects findings by Lode *et al*,^27^ who highlight the importance of integrating personal learning with strategies at the team and system levels.

Participant feedback emphasised the importance of role-relevant RCB content that could be applied in practice. These insights suggest that future RCB programmes should align with professional roles to ensure relevance.^28^ This could be achieved through pre-programme engagement with participants to map session content to professional roles and identify the most relevant RCC tool items for respective staff groups. Participants noted that group discussions and accessible resources supported their engagement and understanding of research. At the same time, the 1 hour format had some limitations, with less time available for discussion and clarification with facilitators. To give participants more time for questions or follow-up, sessions could be extended, or brief drop-ins added after each one. Based on our experience with a face-to-face RCB programme delivered to research interns, fortnightly drop-in sessions facilitated online have complemented teaching and learning.^29^

Participants identified the need for mechanisms to track and apply knowledge and skills over time. This perspective is supported by Hibbert *et al*^30^ in the broader capacity-building literature, who emphasise that sustainable improvement requires a systematic approach, incorporating iterative cycles of learning and development. By treating programmes as ongoing activities with continuous feedback and refinement, organisations can support the longer-term use of newly acquired skills. Likewise, McEvoy *et al*^31^ highlighted the importance of tailored tools for tracking research capacity following RCB endeavours. These findings suggest the necessity of embedding evaluation mechanisms, such as tracking project outcomes and monitoring skill application, into future programme designs to understand longer-term outcomes beyond initial implementation.

### Recommendations for future iterations

Future RCB education programmes using this format at larger scale should consider extending session times to 90 min, and/or adding online drop-in sessions to provide additional support. Attaching research mentors to specific healthcare teams undertaking the RCB programme may help support learning and ongoing engagement in research activity.^32 33^ Overcoming systemic barriers is likely to require logistical support, such as protected research time and alignment with policy. As Gill *et al*^34^ suggest, tailoring session content to fit participants’ roles and organisational goals may enhance relevance and engagement. Lastly, ongoing evaluation should be part of any future programme.^35^

### Study limitations

The small sample in this pilot study from a single organisation in the North West of England limits the transferability of findings to other workforce settings and geographical locations. The organisational context may influence transferability, as organisational culture and infrastructure are recognised as key determinants of research engagement.^36^ Demographic data were not collected, limiting contextualisation by role or professional background. This was deliberate to protect anonymity within the small single-organisation sample. Inferential analysis of matched pre–post questionnaires was not possible; therefore, changes in median scores should be interpreted as indicative only. Lower post-session response rates may also introduce response bias. Qualitative data reflected a single group interview with seven participants and may not capture the perspectives of all programme participants. Finally, the findings should be considered preliminary evidence of feasibility and acceptability of this RCB education delivery method, not its effectiveness. Future research would benefit from larger samples and longitudinal tracking of intended outcomes.

## Conclusions

This pilot study found initial feasibility and acceptability of an online approach to RCB education. The format offers a scalable and practical alternative to face-to-face delivery for a time-constrained health and care workforce, with minimal service disruption. Preliminary improvements in behaviour intention alongside confidence and skill gains suggest the programme may offer benefits beyond access alone. Similar RCB educational approaches may support organisations working towards the research-ready workforce outlined in NHS England policy ambitions. The next stage of research should examine the feasibility of implementation at greater scale, across diverse settings, and consider longer-term outcomes and impact.

## Supplementary material

10.1136/bmjopen-2026-118946 online supplemental file 1

10.1136/bmjopen-2026-118946 online supplemental file 2

## Data Availability

Data are available in a public, open access repository.
