# Supplementary material for: Evaluating the feasibility and acceptability of an online research capacity-building education programme for the health and care workforce: a mixed-methods pilot study
Source: BMJ Open. 2026 Aug 11;16(8):e118946. doi: 10.1136/bmjopen-2026-118946 (PMC13475142; doi:10.1136/bmjopen-2026-118946)
Supplement: Supplementary file 1 [file bmjopen-2026-118946.reviewer_comments.pdf]

PEER REVIEW HISTORY

BMJ Open publishes all reviews undertaken for accepted manuscripts. Reviewers are asked to complete a checklist review form and are provided with free text boxes to elaborate on their assessment. These free text comments are reproduced below.

ARTICLE DETAILS

Title (Provisional)

Evaluating the Feasibility and Acceptability of an Online Research Capacity-Building Education Programme for the Health and Care Workforce: A Mixed-Methods Pilot Study

Authors

Lamont, Scott; Berzins, Kathryn; Aspinall, Georgia; Lowther-Payne, Hayley J; Boland, Paul; Harris, Catherine; Harrison, Joanna; Hirst, Yasemin; Spencer, Joseph; Weldon, Jo Catherine; Watkins, Caroline Leigh

---

VERSION 1 - REVIEW

Reviewer 1

Name Bishop, Jaclyn

Affiliation East Grampians Health Service

Date 14-May-2026

COI No direct conflict however I have experience with a research capacity building program in my local area.

Thank you for submitting this article on an online research capacity building program.

While I appreciate the work that has gone into the development of the program, the evaluation and preparing the manuscript, I feel that the limitations represent a barrier to publication in this journal. Here are my key reasons:

- This was a single site study with a small number of participants.
- The small number of participants combined with a low return rate hampered the ability to draw any conclusions from the surveys [CPD-R].
- The study is framed as mixed methods and the GRAMMS checklist seeks to highlight the integration. However, the lack of paired survey data limits this substantially and the integration is unclear in the results/discussion.
- The discussion highlights how many of the findings from the survey aligned with previous research on this topic. It is unclear therefore why these learnings were not incorporated into the program design at the outset.

My recommendation would be to make the changes that identified through this pilot study and then evaluate the program at a larger scale. This then could make a greater novel contribution to the literature.

Minor feedback includes:

Introduction

- Several broad statements require a reference e.g. 'However, the health workforce may lack the specific knowledge, skills and support to engage in research activities' and 'The healthcare and education sectors have seen staff RCB initiatives as a way to enhance their research capabilities and to support organisational planning and delivery'.
- 'Research capacity building, whilst shaped by individual knowledge, skills, and motivation, must also be considered in the context of broader organisational issues'. Can you be more specific with what 'issues you are referring to'.

Methods

Context and setting

- Can you define what a 'Health Innovation organisation' is? I am not familiar with the term.
- Who designed the materials?
- What were the 'broader organisational research capacity building efforts' referred to?

Participant recruitment

- Who recruited the participants?
- Who undertook consent and what was the process (particularly for the interviews)?

Data collection

- How long after the sessions was the survey offered? Why this timeframe?
- More information is required on the interviewers and their skills, biases (etc). See CoreQ checklist as a guide.

Data analysis

- Who specifically did the quantitative analysis?
- Who specifically did the qualitative analysis? What experience did they have in this approach?

Results

Survey

- Did you collect data on the demographics of the attendees in the training?

- I would suggest avoiding using non-specific terms/phrases like 'modest' and 'larger increases'.

Group interview

- This section is quite long – consider making it more concise to keep the reader engaged.

Discussion:

- The second paragraph suggests that there were known factors that would make such a program more successful, and I'm unclear why the researchers didn't factor that into the program design e.g. 'Participants in this study indicated that for training to be effective, it needs to reflect what participants actually do in their roles. This idea is echoed in the work of Cordrey et al. [20] and Brandenburg et al. [21], who found that content is more effective when tailored to the distinct needs of specialist teams' and 'The ongoing tension between individual and organisational aims found here reflects findings by Lode et al. [22], who highlight the importance of integrating personal learning with strategies at the team and system levels'.
- 'These insights suggest that future RCB programmes should align with professional roles to ensure relevance [23]' → how do you propose to do this?

**Reviewer**	**2**
**Name**	**Peckham, Stephen**
**Affiliation**	**University of Kent, CHSS**
**Date**	**22-May-2026**
**COI**	**None**

This paper aims to evaluate the feasibility and acceptability of an online research capacity-building education programme and explore preliminary outcomes to inform future scale delivery. It was a small study conducted within a single Health Innovation Network in England. The context is important and no detail is given about what the role of the organisation is and how it relates to health services. This is important given the reference by the authors as to this being a key limitation of the study in terms of its generalisability. More detail is particularly needed for international readers, especially as most interest is in building research capacity of healthcare professionals involved in service delivery and the critical importance of organisational perspectives (see for example Boaz et al 2025, Peckham et al 2023). I was surprised that while noting most methods training is delivered face-to-face that there is no broader discussion or reference in the paper to evidence on the method of delivery. There is a substantial literature on on-line learning, including the delivery of

research methods and it is not clear whether this had been drawn on in designing the intervention. Clearer description of context and situating the paper in wider discussions of online learning This would have been useful given the authors argue that

"Online RCB education offers a flexible, scalable alternative to traditional face-to-face RCB programmes. Understanding the feasibility and acceptability of online programmes can inform their future implementation and potential for wider delivery across healthcare settings."

The study is an evaluation of online learning in research methods based on the Research Capacity Development for Impact framework. The intervention consisted of hour-long online sessions focused on 13 of 14 Research Capacity and Culture tool 'Individual' items. They were attended by between 2 and 11 staff members. No detail is given of how many sessions, or which ones were attended by the same staff member. Data collection was limited by the low response rates for post session questionnaires although engagement in the post course discussion group was higher (7/11). Some useful theses have been identified in the paper, and the findings are reported clearly and well. There were positive impacts reported as well as useful feedback helpful in future programme design.

The authors rightly note a number of limitations of the study and how this limits the conclusions that can be drawn from the study. Key findings note the importance of tailoring studies to individual roles which given the study organisation is fairly unique is a distinct limitation of the study. I also not that the discussion focuses on the sustaining of individual engagement in research through tracking rather than a clearer discussion of the method of delivery Points noted in the paper about length of sessions, adding drop-in sessions, system barriers etc are issues already identified within the literature on on-line education. More attention to these would have perhaps provided a more tailored intervention.

While the study was well conducted and reported my own feeling is that the limitations of the context, sample and intervention severely limit the value of the paper for other audiences. I think that a substantial rewrite embedding the study in some more relevant literature would be helpful as well as a clearer description of context and relevance. It might have been helpful to follow up with data on the impact on the organisation and how the programme had impacted it at a later date rather than just an immediate impact assessment given the importance of the organisational context. As it stands the paper produces useful insights but with limited relevance across health services, perhaps adding little in its current form to current knowledge.

References:

Boaz, A., Goodenough, B., Hanney, S. and Soper, B., 2024. If health organisations and staff engage in research, does healthcare improve? Strengthening the evidence base through systematic reviews. Health Research Policy and Systems, 22(1), p.113.

Peckham S, Zhang W, Eida T, Hashem F, Kendall S (2023), "Research engagement and research capacity building: a priority for healthcare organisations in the UK". J Health Organ Manag, Vol. 37 No. 3 pp. 343–359, doi: https://doi.org/10.1108/JHOM-12-2021-0436

VERSION 1 - AUTHOR RESPONSE

Reviewer: 1
Dr. Jaclyn Bishop, East Grampians Health Service
Thank you for submitting this article on an online research capacity building program. While I appreciate the work that has gone into the development of the program, the evaluation and preparing the manuscript, I feel that the limitations represent a barrier to publication in this journal. Here are my key reasons:

This was a single site study with a small number of participant. The small number of participants combined with a low return rate hampered the ability to draw any conclusions from the surveys [CPD-R:

Response: Thanks, we agree that the small sample and low number of matched pre–post CPD-R responses limit the conclusions that can be drawn from the survey data. This is acknowledged in the Results and Study limitations sections, where we state that inferential analysis was not possible and that the findings are indicative only. We have further clarified this in the **'Results'** section relating to CPD-R findings adding the following statement:
Accordingly, the CPD-R findings are presented descriptively and should be interpreted as exploratory.

The study is framed as mixed methods and the GRAMMS checklist seeks to highlight the integration. However, the lack of paired survey data limits this substantially and the integration is unclear in the results/discussion:

Response: Thanks, we have clarified the nature of the mixed-methods adding the following sentence into the **'Data Analysis'** section

CPD-R data offered preliminary, exploratory insight into behavioural intention. Qualitative findings addressed both feasibility and acceptability, including practical and logistical factors relevant to delivery. These provided complementary rather than convergent evidence across the study.

The discussion highlights how many of the findings from the survey aligned with previous research on this topic. It is unclear therefore why these learnings were not incorporated into the program design at the outset:

Response: Thanks, we have added the following statement to the '**Intervention'** section of '**Methods'** to clarify the context and position of this specific intervention for the reader:

The format was developed with the partner organisation around protected weekly CPD time and constraints on releasing staff for face-to-face training. While the RCC tool has previously been used to assess research capacity and identify training needs, its use to structure the content of a delivered education programme is, to our knowledge, novel.

My recommendation would be to make the changes that identified through this pilot study and then evaluate the program at a larger scale. This then could make a greater novel contribution to the literature:

Response: Thanks, we agree that the refined programme should next be evaluated at larger scale. This pilot was undertaken specifically to assess feasibility and acceptability and to inform the design of a subsequent effectiveness evaluation**.**

introduction - Several broad statements require a reference e.g. 'However, the health workforce may lack the specific knowledge, skills and support to engage in research activities' and 'The healthcare and education sectors have seen staff RCB initiatives as a way to enhance their research capabilities and to support organisational planning and delivery'.:

Response: Thanks, the following citations have been added to support respective statements:

Matheson M, Skinner IW, Vehagen A, et al. Barriers and enablers of primary healthcare professionals in health research engagement: a systematic review of qualitative studies. Nursing & Health Sciences. 2025;27(1):e70022.

Tajuria G, Dobel-Ober D, Bradley E, et al. Evaluating the impact of the supporting the advancement of research skills (STARS) programme on research knowledge, engagement and capacity-building in a health and social care organisation in England. BMC Medical Education. 2024;24(1):126

'Research capacity building, whilst shaped by individual knowledge, skills, and motivation, must also be considered in the context of broader organisational issues'. Can you be more specific with what 'issues you are referring to':

Response: Thanks, the statement which follows highlight some of these. We have added in 'For example' as a segue to these.

Methods Context and setting - Can you define what a 'Health Innovation organisation' is? I am not familiar with the term:

Response: Thanks, we have added the following context to this section:

The organisation is one of 15 regional Health Innovation Networks established by NHS England to connect the NHS, academia, industry, local authorities and the third sector, supporting the adoption and spread of innovation across health and social

care.

Who designed the materials?

Response: Thanks, the following statement concluding '**Context and Setting'** section has been revised for clarity:

Sessions were designed and facilitated by study authors selected for relevant methodological expertise from within the Applied Research Collaboration North West Coast.

What were the 'broader organisational research capacity building efforts' referred to?

Response: Thanks, the statement was potentially misleading as written and now reads:

An online RCB education programme was designed and delivered to staff to develop foundational research skills as part of organisational efforts to strengthen research capacity.

Participant recruitment - Who recruited the participants?

Response: Thanks, recruitment statement revised to following for additional clarity:

Participants were recruited via internal staff email, circulated by a member of the organisation's evaluation team.

Who undertook consent and what was the process (particularly for the interviews)?

Response: Thanks, please see response to editor comment regarding consent which now addresses this.

Data collection - How long after the sessions was the survey offered? Why this timeframe?

Response: Thanks, we have added the following context to the '**Data Collection'** section:

Post-session surveys remained open for four weeks, with participants asked to complete them 2–4 weeks after each session to allow time for reflection while retaining session proximity.

More information is required on the interviewers and their skills, biases (etc). See CoreQ checklist as a guide:

Response: Thanks, the relevant statement regarding interviewers has been amended in the **'Data Collection'** section to the following:

The group interview was conducted online via Microsoft Teams by two researchers from an applied health research team (GA, HJL-P) who were undertaking postgraduate research degrees, had experience in qualitative methods, and were independent of the education delivery. The interview was recorded and transcribed for analysis.

Data analysis - Who specifically did the quantitative analysis? Who specifically did the qualitative analysis? What experience did they have in this approach?

Response: Thanks, relevant analyses components are now initialled. Experience is addressed via the response to previous comment and additional context for first author in **'Data Analysis'** opening statement.

Results Survey - Did you collect data on the demographics of the attendees in the training?

Response: Thanks, demographic data were not collected to protect anonymity. We have added the following statement to the **'Study limitations'** section:

Demographic data were not collected, limiting contextualisation by role or professional background. This was a deliberate to protect anonymity within the small single-organisation sample.

I would suggest avoiding using non-specific terms/phrases like 'modest' and 'larger increases':

Response: Thanks, in response to an earlier comment, we added a statement preceding these findings clarifying that the CPD-R results are presented descriptively and should be interpreted as exploratory. Within this context, the descriptive terms are retained to guide readers towards patterns in Table 2 rather than to imply inferential conclusions.

Group interview - This section is quite long – consider making it more concise to keep the reader engaged:

Response: Thanks, the closing synthesis paragraphs under each NPT theme have been removed, as these largely restated content already evident from the subtheme commentary and quotations.

Discussion: - The second paragraph suggests that there were known factors that would make such a program more successful, and I'm unclear why the researchers didn't factor that into the program design e.g. 'Participants in this study indicated that for training to be effective, it needs to reflect what participants actually do in their roles. This idea is echoed in the work of Cordrey et al. [20] and Brandenburg et al. [21], who found that content is more effective when tailored to the distinct needs of specialist teams' and 'The ongoing tension between individual and organisational aims found here reflects findings by Lode et al. [22], who highlight the importance of integrating personal learning with strategies at the team and system levels'

Response: Thanks, as noted in earlier response, the programme was designed with the partner organisation around protected CPD time and staff-release constraints, and structured using RCC Tool items. The cited literature contextualises findings from this feasibility and acceptability pilot rather than indicating these factors were overlooked. The refinements identified will inform a later effectiveness evaluation.

'These insights suggest that future RCB programmes should align with professional roles to ensure relevance [23]' ◊ how do you propose to do this:

Response: Thanks, we have added the following statement:

This could be achieved through pre-programme engagement with participants to map session content to professional roles and identify the most relevant RCC tool items for respective staff groups.

Reviewer: 2
Prof. Stephen Peckham, University of Kent, LSHTM
Comments to the Author:
This paper aims to evaluate the feasibility and acceptability of an online research capacity-building education programme and explore preliminary outcomes to inform future scale delivery. It was a small study conducted within a single Health Innovation Network in England. The context is important and no detail is given about what the role of the organisation is and how it relates to health services. This is important given the reference by the authors as to this being a key limitation of the study in terms of its generalisability. More detail is particularly needed for international readers, especially as most interest is in building research capacity of healthcare professionals involved in service delivery and the critical importance of organisational perspectives (see for example Boaz et al 2025, Peckham et al 2023:

Response: Thanks, in line with a Reviewer 1 comment, we added a brief description in the **'Context and Setting'** subsection clarifying the role of Health Innovation organisations in connecting health services, academia, industry and local authorities to support innovation across health and social care.

We have also acknowledged in the **'Study Limitations'** section that organisational context may influence transferability and research engagement, and supported this statement.

I was surprised that while noting most methods training is delivered face-to-face that there is no broader discussion or reference in the paper to evidence on the method of delivery. There is a substantial literature on on-line learning, including the delivery of research methods and it is not clear whether this had been drawn on in designing the intervention. Clearer description of context and situating the paper in wider discussions of online learning This would have been useful given the authors argue that

"Online RCB education offers a flexible, scalable alternative to traditional face-to-face RCB programmes. Understanding the feasibility and acceptability of online programmes can

inform their future implementation and potential for wider delivery across healthcare settings."

> **Response:** Thanks, we have strengthened the discussion by situating the study within the broader online learning literature, adding the following to the second 2nd para of **'Discussion'** section**:**
>
> *Online delivery of research methods education can support research skill development and may improve accessibility for learners unable to attend in person [Asghar et al., 2023]. More broadly, online learning for healthcare professionals may achieve learning outcomes comparable to face-to-face approaches, while offering flexibility around professional commitments [Aryee et al., 2024; Richmond et al., 2017]*

The study is an evaluation of online learning in research methods based on the Research Capacity Development for Impact framework. The intervention consisted of hour-long online sessions focused on 13 of 14 Research Capacity and Culture tool 'Individual' items. They were attended by between 2 and 11 staff members. No detail is given of how many sessions, or which ones were attended by the same staff member:

> **Response:** Thanks, cross-session attendance was not tracked, as sessions were designed to be accessed flexibly. This has been clarified in the '**Participant Recruitment'** section with the following addition:
>
> *Sessions were available to all eligible staff, and individual attendance across sessions was not tracked, as participation in one session was not a prerequisite for another.*

The authors rightly note a number of limitations of the study and how this limits the conclusions that can be drawn from the study. Key findings note the importance of tailoring studies to individual roles which given the study organisation is fairly unique is a distinct limitation of the study. I also not that the discussion focuses on the sustaining of individual engagement in research through tracking rather than a clearer discussion of the method of delivery Points noted in the paper about length of sessions, adding drop-in sessions, system barriers etc are issues already identified within the literature on on-line education. More attention to these would have perhaps provided a more tailored intervention.

> **Response:** Thanks, the points raised are addressed through revisions made in response to earlier comments. The Discussion now explains how future iterations could use pre-programme engagement to map content to professional roles, while the Study limitations section acknowledges that the specialist organisational context may affect transferability. The Discussion has also been strengthened to situate the findings within the broader online learning literature, including evidence on accessibility, flexibility and learning outcomes for working professionals. Finally, the Methods clarifies that the programme design was shaped by organisational constraints and involved a novel application of the RCC Tool.

While the study was well conducted and reported my own feeling is that the limitations of the context, sample and intervention severely limit the value of the paper for other audiences. I think that a substantial rewrite embedding the study in some more relevant literature would be helpful as well as a clearer description of context and relevance.

> **Response:** Thanks, we acknowledge the points raised but consider the study to retain value as a feasibility and acceptability evaluation of a novel online RCB approach. We believe the revisions further strengthen its relevance to a wider audience.

It might have been helpful to follow up with data on the impact on the organisation and how the programme had impacted it at a later date rather than just an immediate impact assessment given the importance of the organisational context. As it stands the paper produces useful insights but with limited relevance across health services, perhaps adding little in its current form to current knowledge.

> **Response:** This pilot was designed to assess feasibility and acceptability rather than organisational impact. We agree that longer-term organisational follow-up would be valuable and intend to address this in future effectiveness studies. We consider the study to add to current knowledge as, to our knowledge, the first published evaluation of a programme whose session content was directly structured around the RCC Tool.

References:
Boaz, A., Goodenough, B., Hanney, S. and Soper, B., 2024. If health organisations and staff engage in research, does healthcare improve? Strengthening the evidence base through systematic reviews. Health Research Policy and Systems, 22(1), p.113.
Peckham S, Zhang W, Eida T, Hashem F, Kendall S (2023), "Research engagement and research capacity building: a priority for healthcare organisations in the UK". J Health Organ Manag, Vol. 37 No. 3 pp. 343–359, doi: https://doi.org/10.1108/JHOM-12-2021-0436

VERSION 2 - REVIEW

**Reviewer**	**2**
**Name**	**Peckham, Stephen**
**Affiliation**	**University of Kent, CHSS**
**Date**	**24-Jul-2026**
**COI**	

I appreciate the detail that the authors have gone to in their response to reviewers comments. There has been a clear revision of the paper to strengthen and make clearer context, methods and discussion. While it remains a small study in a Health Innovation Network with a number of limitations I feel the revisions make the purpose and outcomes of the study more useful. They acknowledge the need to follow up this approach. On this basis I am happy to support publication.
