# Supplementary material for: Evaluating the feasibility and acceptability of an online research capacity-building education programme for the health and care workforce: a mixed-methods pilot study
Source: BMJ Open. 2026 Aug 11;16(8):e118946. doi: 10.1136/bmjopen-2026-118946 (PMC13475142; doi:10.1136/bmjopen-2026-118946)
Supplement: Supplementary file 2 [file bmjopen-16-8-s001.docx]

**Supplementary file 1. RCB education sessions**

| **RCC tool Individual item / Education session (n=attendees)** | **Content overview** |
| --- | --- |
|  |  |
| Introduction – Demystifying research (n=10) | - Overview of Applied Research Collaboration NWC  - Overview of Implementation and Capacity Building Team  - Research – context & myths  - Research Capacity Building – what & why  - What’s coming up  - ReCaB Study – Evaluations |
| Finding relevant literature (n=10) | - Planning a search  - Databases  - Search tips  - Doing a search on ESBCO  - Where to access resources |
| Critically reviewing the literature (n=2) | - What is Critical appraisal  - Why do Critical appraisal  - Key elements of bias in research studies  - Critical appraisal process  - Selecting and using frameworks for critical appraisal in both quantitative and qualitative studies |
| Using a computer referencing system (e.g. Endnote) (n=8) | - Why and when to reference  - Overview of referencing tool EndNote  - Downloading and organising references.  - Inserting citations and bibliographies in Microsoft Word  **-** Endnote tips |
| Writing a research protocol (n=8) | - Purpose and structure of a research protocol  - Key components: background, rationale, and theoretical framework  - Defining research questions, objectives, and methodology  - Ethical and regulatory considerations  - Appendices: supporting documents (e.g., consent forms, participant information sheets)  - Writing tips: clarity, conciseness, and logical structure  - Collaboration and role definition in the research process |
| Submitting an ethics application (n=6) | - Overview of research governance via the NHS Health Research Agency  - Identifying and mitigating ethical issues  - Preparing essential documents  - Role of NHS Trust Research & Development department  - Personal permissions |
| Designing questionnaires (n=6) | - Purpose and goal setting for questionnaires  - Consideration of existing validated questionnaires  - Stages in questionnaire design  - Key components: information sheet, screening questions, and types of data  - Question types: closed and open questions  - Writing effective, unbiased questions  - Question order and its impact on responses  - Strategies to increase response rate  - Pilot testing for clarity and effectiveness  - Ensuring validity and reliability in questionnaire design |
| Collecting data (e.g. surveys, interviews) (n=11) | - Understanding research data  - Different types of research data  - Theoretical/Conceptual Frameworks  - Data collection methods  - Sampling considerations  - Ethical considerations  - Data protection |
| Using computer data management systems (SPSS/NVIVO) (n=7) | - Data Management Systems in Research  - Qualitative Data Management & Analysis Software  - NVivo Quick Demo  - Quantitative Data Management & Analysis Software  - SPSS Quick Demo  - Ethical Considerations & Data Protection |
| Analysing qualitative research data (n=9) | - Preparing data: transcription methods and considerations  - Coding: defining and assigning labels to data (deductive and inductive approaches)  - Thematic analysis: six stages from familiarising to reporting  - Developing themes: identifying and clustering codes  - Iterative analysis: refining themes and re-coding as needed  - Using qualitative analysis software |
| Analysing quantitative research data (n=5) | - Summary statistics: mean, standard deviation, median, and interquartile range  - Interpreting results: confidence intervals and p-values  - Statistical significance vs. clinical significance  - Key statistical tests: chi-square, t-test, ANOVA, correlation, and regression  - Practical application with example datasets  - Considerations for effective interpretation of quantitative data |
| Writing a research report (n=6) | - Purpose and importance of research reports  - Structure of a research report (IMRAD format) - Title and abstract; Introduction and literature review; Methodology; Results; Discussion; Conclusions; References; Appendices  - Tips for writing, revising, and finalising reports |
| Writing for publication in peer-reviewed journals (n=5) | - Why Publish?  - Authorship – who and what  - Where to Publish  - Style & Formatting  - Navigating the submission process  - Navigating the review process  - Manuscript Decisions and options  - You’re Published – So What Next? |
| *Supporting less experienced researchers (n=4) | - Benefits of fostering supportive research environments  - Key mentorship practices: visibility, collaboration, and knowledge-sharing  - Encouraging critical thinking and continuous reading  - Recognising and celebrating achievements  - Establishing communities of practice and journal clubs  - Offering training and development programs  - Fostering innovative research cultures and enhancing resource access  - Strategies for developing collaborative networks |
| **Understanding Implementation Science (n=6) | - What is implementation science and why it is important  - Understanding research methods used in implementation science  - Considering complexity of carrying out implementation research  - Understanding the use of theory in implementation science  - Considering differences and uses of different theoretical models |
| **Completing a Health Inequality Assessment Tool (n=9) | - What is a HIAT?  - Mapping inequalities relative to your research  - Integrating equity issues into research questions  - Designing and conducting research sensitive to inequalities  - Prioritising findings relevant to action on inequalities in reporting & dissemination  - Principles and practice in equity sensitive research  - Using a HIAT during the research process |

*Amended from Holden et al (2014)

** Additional items added to Holden et al’s (2014) framework

N.B. ‘Securing Research Funding’ (Holden et al., 2014) omitted from education delivery as the organisation is commissioned to undertake projects
