# Supplementary material for: Evaluating the feasibility and acceptability of an online research capacity-building education programme for the health and care workforce: a mixed-methods pilot study
Source: BMJ Open. 2026 Aug 11;16(8):e118946. doi: 10.1136/bmjopen-2026-118946 (PMC13475142; doi:10.1136/bmjopen-2026-118946)
Supplement: Supplementary file 3 [file bmjopen-16-8-s002.docx]

**Objective: (insert CPD behavior/objective here and repeat as necessary)**

Please answer each of the following questions by indicating the number that best describes your opinion about the behavior indicated. Some of the questions may appear to be similar, but they do address somewhat different aspects of the behavior

| 1. | I intend to *(behavior)*. | **Strongly disagree** | | |  |  | **Strongly agree** | |
| --- | --- | --- | --- | --- | --- | --- | --- | --- |
|  |  | 1 | 2 | 3 | 4 | 5 | 6 | 7 |
| 2 | To the best of my knowledge, the percentage of my colleagues who *(behavior)* is: | **0-20** | **21-40** |  | **41-60** | **61-80** |  | **81-100** |
|  |  | 1 | 2 |  | 3 | 4 |  | 5 |
| 3 | I am confident that I could *(behavior)* if I wanted to. | **Strongly disagree** | | |  |  | **Strongly agree** | |
|  |  | 1 | 2 | 3 | 4 | 5 | 6 | 7 |
| 4 | *(behavior)* is the ethical thing to do. | **Strongly disagree** | | |  |  | **Strongly agree** | |
|  |  | 1 | 2 | 3 | 4 | 5 | 6 | 7 |
| 5 | For me, *(behavior)* would be: | **Extremely difficult** | | |  |  | **Extremely easy** | |
|  |  | 1 | 2 | 3 | 4 | 5 | 6 | 7 |
| 6 | Now think about a co‐worker whom you respect as a professional, in your opinion, does he/she *(behavior)?* | **Never** | | |  |  | **Always** | |
|  |  | 1 | 2 | 3 | 4 | 5 | 6 | 7 |
| 7 | I plan to *(behavior)*. | **Strongly disagree** | | |  |  | **Strongly agree** | |
|  |  | 1 | 2 | 3 | 4 | 5 | 6 | 7 |
| 8 | Overall, I think that for me *(behavior)* would be: | **Useless** | | |  |  | **Useful** | |
|  |  | 1 | 2 | 3 | 4 | 5 | 6 | 7 |
| 9 | Most people who are important to me in my profession *(behavior)*. | **Strongly disagree** | | |  |  | **Strongly agree** | |
|  |  | 1 | 2 | 3 | 4 | 5 | 6 | 7 |
| 10 | It is acceptable to *(behavior)*. | **Strongly disagree** | | |  |  | **Strongly agree** | |
|  |  | 1 | 2 | 3 | 4 | 5 | 6 | 7 |
| 11 | I have the ability to *(behavior)***.** | **Strongly disagree** | | |  |  | **Strongly agree** | |
|  |  | 1 | 2 | 3 | 4 | 5 | 6 | 7 |
| 12 | Overall, I think that for me *(behavior)* would be: | **Harmful** | | |  |  | **Beneficial** | |
|  |  | 1 | 2 | 3 | 4 | 5 | 6 | 7 |
